# Cortical Source Analysis of the Face Sensitive N290 ERP Component in Infants at High Risk for Autism

**DOI:** 10.3390/brainsci12091129

**Published:** 2022-08-25

**Authors:** Maggie W. Guy, John E. Richards, Jane E. Roberts

**Affiliations:** 1 Department of Psychology, Loyola University Chicago, Chicago, IL 60660, USA; 2 Department of Psychology, University of South Carolina, Columbia, SC 29208, USA

**Keywords:** autism spectrum disorder, cortical source analysis, event-related potentials, face processing

## Abstract

Appropriate head models for cortical source analysis were investigated and applied to source analyses examining the neural bases of the face-sensitive N290 event-related potential (ERP) component in infants at high risk for autism spectrum disorder (ASD). This included infant siblings of children with ASD (ASIBs) and infants with fragile X syndrome (FXS). First, alternative head models for use with ASIBs and FXS were investigated. Head models created from the infant’s own MRI were examined in relation to five head models based on average MRI templates. The results of the head model comparison identified group-specific (i.e., ASIB or FXS) head models created from a large collection of structural MRIs as the best substitution for the head model created from the participant’s own structural MRI. Second, the cortical source analysis was completed on N290 data collected from a previous study to investigate brain areas associated with face sensitive ERP responses. Participants’ own MRIs were used for head models when available, and the group-specific head model was used when the participants’ own MRIs were not available. The results provide evidence for unique patterns of neural activation during face processing across infants at high and low risk for ASD and across etiologically distinct high-risk groups. All infants demonstrated greater activation to faces than toys in brain areas most associated with specialized face processing. Infants with FXS displayed higher levels of activation to faces across all areas analyzed, while ASIBs show more muted levels of activation. Overall, the results of the current study demonstrate the importance of group-specific head models for accurate cortical source analysis in infants at high risk for ASD. This also allows for further research on early distinctions in brain function based on risk status.

## 1. Introduction

Research using event-related potentials (ERPs) has shown that atypical neural responses are present in infants at elevated risk for autism spectrum disorder (ASD) prior to the production of behavioral symptoms e.g., [1,2,3,4,5,6]. Our own research has identified atypical patterns of responses at the N290 and Negative central (Nc) ERP components to visual stimuli in two groups of infants at high risk for ASD. This includes infant siblings of children with autism (ASIBs) and infants with fragile X syndrome (FXS) [4]. These infant groups are at elevated risk for ASD. Approximately 20% of ASIBs [7,8,9] and 60% of infants with FXS [10] will receive an ASD diagnosis. These rates are much higher than the rate of 1.9% in the general population [11]. In the current study, we utilized cortical source analysis to better understand the neural bases of the N290 response in infants at high risk for ASD. The N290 is closely linked with face processing in infancy, e.g., [12,13,14], and atypical face processing is one of the most frequently observed forms of atypical visual attention in ASD, e.g., [15,16,17]. Diagnosis with ASD typically occurs in early childhood; investigating the N290 and its cortical sources in infancy can provide important information about the development of face processing in autism and inform early markers of ASD risk. Cortical source analysis combines electrophysiological data measured on the scalp with a model of the head to estimate likely neural sources within the brain. It is an ideal method for localizing brain activity in infants, as it is safe and possesses fewer task demands than alternative methods, such as intracranial EEG recording or fMRI. This is the first study to examine neural generators of ERP responses, including the N290, in infants at an elevated risk for ASD.

### 1.1. The Infant N290

Examination of the infant N290 ERP component may be especially informative to the development of ASD. The amplitude of the N290 has been associated with the presentation of symptoms of ASD in childhood [18]. Specifically, Shephard and colleagues [18] found that greater N290 amplitudes to faces versus noise at 7 months of age were associated with more severe social-communication impairment at 7 years of age in ASIBs, but not low-risk, TD participants. The N290 reflects developing face specialization, e.g., [12,13], and is believed to be a precursor to the adult N170 component [14,19,20,21]. The N290 is characterized by a negative peak occurring approximately 290 ms after stimulus onset at lateral posterior electrode sites, e.g., [12,13]. Greater N290 amplitude occurs to faces compared with other classes of stimuli [12,13,14]. Discrimination of face and non-face stimuli at the N290 has been observed in studies including ASIBs [4,6] and infants with FXS [4]. Guy et al. [4] measured ERP responses from 12-month-old ASIBs, infants with FXS, and typically developing low-risk control (LRC) infants in response to photographs of the participant’s mother’s face, a stranger’s face, the participant’s favorite toy, and a novel toy. Infants with FXS demonstrated an enhanced N290 response to their mother’s face and a muted response to the novel toy, which was reflected in significantly greater N290 amplitude to familiar than novel stimuli. Only infants with FXS demonstrated unique N290 responses based on stimulus familiarity. Their responses were also greater than ASIB and LRC infants’ responses to familiar and novel stimuli. Key and Stone [22] and Luyster and colleagues [23] conducted studies in which ASIBs and LRC infants were presented with pictures of their mother’s face and a stranger’s face. Key and Stone [22] observed a greater amplitude N290 response to the mother’s face than a stranger’s face for both 9-month-old LRC infants and ASIBs. However, Luyster et al. [23] observed only a marginally significant difference in N290 amplitude for LRC infants, such that a more negative N290 was displayed to the stranger’s face than the mother’s face. ASIBs did not discriminate faces based on N290 amplitude [23]. This effect has not been replicated in other studies of typically developing infants [13,24,25,26] and ASIBs [6], and variability in results may be influenced by stimuli, participant age, and ERP processing methods. Most studies have included both face and non-face stimuli, while two of the studies identifying effects of stimulus familiarity on N290 amplitude have only included familiar and novel face stimuli [22,23]. More work is needed to better understand the role of familiarity in the infant N290.

### 1.2. Cortical Source Analysis

Cortical source analysis can be used to understand underlying neural mechanisms contributing to N290 differences observed between infants with FXS and ASIBs at the scalp. It has been proposed that greater amplitude responses in FXS are reflective of synaptic hyperresponsivity [27], while muted responses in ASIBs may support hyporesponsivity. Sources of the N290 have been investigated in typically developing infants [12,13,28,29]. The fusiform gyrus has been identified as the generator of the N290 in recent work examining developing face specialization in 3- to 12-month-old infants [12,13]. The middle fusiform gyrus showed greater activation to faces than toys, with a peak in activation that was consistent with the peak of the N290. Other brain areas (e.g., anterior fusiform gyrus, and parahippocampal gyrus) showed larger activity to faces than objects during the time window of the N290 but did not show the peak in activation at the peak of the N290 ERP component. Increased activation is not sufficient to conclude that an ROI is a source of a component. The parahippocampal gyrus demonstrates a strong linear increase in activation across the time window of the N290 and may contribute to the later-occurring P400 component.

The N290 is believed to be a precursor to the adult N170 ERP component [12,13,14] and these results are consistent with cortical source analysis results for the N170. The N170 is closely associated with face processing in adults, and greater amplitude N170 is observed in response to faces compared with non-face stimuli, including houses and other objects [30,31,32,33,34,35,36]. A number of sources have been identified as neural contributors to the N170 (for summary see 37 supplemental information). However, the area most consistently identified as the primary source of the N170 is the fusiform gyrus (specifically the middle and posterior fusiform gyrus) [37,38,39,40]. The role of the fusiform gyrus in face processing has also been supported in research measuring both ERP and fMRI responses to face stimuli in adult participants [37,41,42,43]. Decreased fusiform gyrus activation in individuals with FXS has been reported [44], which could indicate that enhanced activation during face processing observed in infants with FXS is not due to increased activation in sources typical of the N290 and N170.

### 1.3. Head Model Selection

The optimal approach for electrical source analysis involves using realistic head models based upon individual participants’ own MRIs [12,13,45]. However, it is often not feasible to collect MRIs from each participant given the high cost and issues with participant compliance. An alternative is to use head models derived from average MRI templates. Several EEG/ERP source analysis methods use the templates derived from the MNI template (Montreal Neurological Institute) [46,47]. Age-specific MRI templates based on infants or children should be better than those based on adults [48], for review [49]. Rapid brain growth in early development is associated with variability in brain size, shape, and tissue, even within close age ranges [50,51,52,53,54,55]. Recent advances have made age-specific MRI templates available for use from birth through late adulthood [53,54,55,56,57,58].

It may be important to consider known neural and neurophysiological differences across participant subgroups. Infants at high risk for ASD demonstrate atypical patterns of brain development. Studies have found that children later diagnosed with ASD showed greater than average head circumference by 12 to 24 months of age, believed to reflect overgrowth of the brain [59,60,61,62]. Hyperexpansion of the surface of the cortex was observed between 6 and 12 months of age in ASIBs that demonstrated brain overgrowth at 12 to 24 months of age and were later diagnosed with ASD [63]. Additionally, both children with ideopathic ASD and children with FXS have shown greater volume than typically developing (TD) children, and areas of enlargement differed across groups [64]. These differences may be present in infancy. If so, this supports the use of separate head models for ASIBs and infants with FXS. The differences in brain development between these groups could affect the accuracy of head models used in source analysis.

### 1.4. The Current Study

The current study had two goals. First, we investigated alternative head models for use with etiologically distinct infants at high risk for ASD (i.e., ASIBs and FXS). Realistic head models created from participant’s own MRIs are considered to be the gold standard for use with cortical source analysis e.g., [65,66]. We tested the similarity of head models created from the infant’s own MRI with head models based on average MRI templates. The average MRI template head models came from study-specific average head models made from the two risk groups (SS-ASIB, SS-FXS), group-specific average head models created with MRIs from the Infant Brain Imaging Study (IBIS) database (IBIS-ASIB or IBIS-FXS), an average head model created from 12-month-old typically developing infants (12-mo-TD), an average head model created from 12-year-old TD children (12-yr-TD), and an average head model created from TD adults (Adult-TD). These alternative head models were selected because researchers may reasonably consider the use of head models derived from MRIs of those sharing the same risk for or diagnosis with neurodevelopmental disorders (i.e., SS and IBIS head models), MRIs of infants of the same age (i.e., 12-mo-TD head model), and MRIs that are convenient to access (i.e., 12-yr-TD and Adult-TD head models). Comparing the similarity of the results based on the individual MRIs with those based on the average MRI templates will aid in the selection of appropriate average MRI templates.

Second, we investigated brain areas associated with face-sensitive ERP responses in high-risk infants through the use of cortical source analysis using a pipeline established for pediatric research see [48]. Results from the head model comparison contributed to this goal. Individual head models derived from participant’s own MRIs were used when available, and the average head model that provided the best substitute for the own MRI was used when the own MRI was not available. The N290 data from a previous study [4] were analyzed to address whether differences in scalp activation to social stimuli across high- and low-risk infant groups are due to differences in areas of brain activation, or differences in amplitude of activation. We focused on the N290 ERP component and its known cortical sources [12,13,37], comparing the cortical source of the N290 ERP component for ASIB, FXS, and LRC infants at 12 months of age. This work will contribute to the better understanding of neural sources responsible for different patterns of ERP activity observed across these distinct groups of infants at elevated risk for autism.

## 2. Materials and Methods

### 2.1. Participants

Fifty-seven 12-month-old infants participated in the current study, including 21 ASIBs (*N* = 18 males), 15 infants with FXS (*N* = 8 males), and 21 typically developing, LRC infants (*N* = 16 males). Only infants at high risk for ASD that also completed the structural MRI recording (i.e., 8 ASIBs and 11 infants FXS) were included in the head model comparison. All participants were included in the cortical source analysis of the N290.

### 2.2. Procedure

#### 2.2.1. EEG Recording

Upon arrival to the Infant Development Lab at the University of South Carolina, infants were fitted for an EGI Hydrocel Geodesic Sensor Net. They were positioned on their parent’s lap in front of a monitor for stimulus presentation. A video camera was positioned above the monitor to record participants’ looking behavior. An experimenter controlled stimulus presentation in an adjacent room. ERP stimuli were presented for 500 ms and included a photograph of the participant’s mother’s face, the previous participant’s mother’s face, the participant’s favorite toy, and the previous participant’s favorite toy. Dynamic audiovisual clips of Sesame Street were presented at the beginning of each trial block and whenever the infant appeared distracted. There was a variable inter-trial interval of 500–1500 ms between stimulus presentations. For further details on the EEG recording, see Guy et al. [4].

#### 2.2.2. Magnetic Resonance Imaging

Infants’ MRI data were collected in a Siemens Medical Systems 3T Trio scanner during natural sleep (without sedation) within 30 days of their 12-month-old birthday. The scanner is housed in the McCausland Center for Brain Imaging at Palmetto Health Hospital in Columbia, SC. Members of the Infant Development Lab have mastered a procedure for the collection of infant MRIs by allowing the infant to fall asleep in the scanner room, then fitting them with infant earplugs and headphones, and gently situating them on the MRI bed surrounded by foam pads to keep still throughout the scan. An MRI technician, parent of the infant, and radiology nurse remain in the MRI room during the scan, which lasts approximately 15 min and includes a localizer sequence, a 3D sagittal T1-weighted scan, and a T2-weighted scan. The field of vision for the scans included the top of the head down to the neck.

An additional 77 structural MRIs were obtained from the Infant Brain Imaging Study (IBIS). Fifty-three of the scans were collected from 12-month-old ASIBs and 24 scans were collected from 12-month-old infants with FXS. Of the 53 scans acquired from infant ASIBs, 27 were collected from infants later diagnosed with ASD and 26 were from infants that were not diagnosed with ASD. The IBIS participants with FXS were not all tested for ASD, and therefore we do not have outcomes for their data. All 53 of the scans collected from infant ASIBs were used in creation of the IBIS-ASIB head model and all 24 of the scans collected from infants with FXS were used in creation of the IBIS-FXS head model. Infants that contributed scans to IBIS did not participate in the ERP or MRI portions of this study.

### 2.3. Data Processing

**Figure 1:**
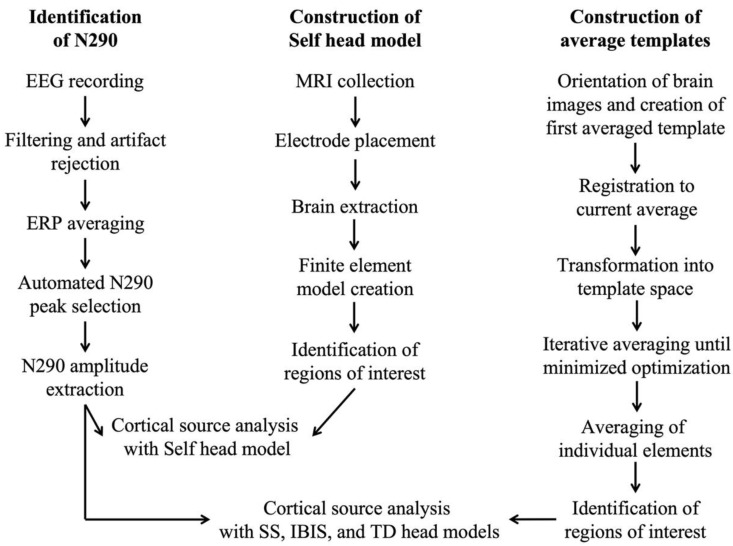
The workflow for the processing of electroencephalogram (EEG), event-related potential (ERP), and magnetic resonance imaging (MRI) data is presented, highlighting major steps in the processing stream. The “Construction of Self head model” stream was used for cortical source analysis of the N290 with the participant’s own MRI. The “Construction of average template” stream was used for cortical source of the N290 with the Infant Brain Imaging Study (IBIS), study-specific (SS), and typically developing (TD; i.e., 12-mo-TD, 12-yr-TD, and Adult-TD) averaged head models.

Figure 1 provides an overview of major steps in the data processing streams for the EEG and MRI data. The imaging data was processed at the individual (i.e., head models were created from individual participant’s MRIs for use with their data) and group (i.e., head models were created from averages of groups of MRIs) level. Steps in the data processing workflow are covered in greater detail in the following paragraphs.

#### 2.3.1. MRI File Preparation

The MRI files were processed using a procedure similar to that previously described by Richards and colleagues [53,54,56]. The brains were extracted from the whole-head MRI volume using FSL brain extraction tools. Each brain extraction was visually inspected for accuracy and adjusted as necessary using the bet2 variables (e.g., fractional intensity threshold, center of gravity, and starting sphere size) to obtain a well-formed brain volume [67].

Realistic head models were created from the structural MRIs. The materials within individual participants’ heads were identified and assigned a relative conductivity. The MRIs were segmented into gray matter, white matter, non-neural brain tissue, cerebrospinal fluid, skull, scalp, eyes, nasal cavity, and areas outside the scalp in the lower part of the head (consisting of muscle and spinal column). This segmentation was used to create a finite element method (FEM) model for each MRI. FEM models consider the geometry and conductivity of the materials within the head at the level of the voxel. The use of FEM models, including the segmentation of these brain materials, has been recently empirically validated [68]. Specific conductivity values are assigned to each material in the FEM model for consideration during cortical source analysis. Conductivity values of each segment are presented in Table 1 as follows: 0.35 S/m scalp, 0.0132 S/m skull, 1.79 S/m cerebrospinal fluid, 0.2 S/m white matter, 0.33 S/m gray matter, 0.33 S/m dura, 0.35 S/m muscles, 0.5 S/m eyes, and 0.0048 S/m nasal cavity.

#### 2.3.2. Construction of Group- and Study-Specific Templates

Average head models were created as “study-specific MRI templates” from the participants in the risk groups (SS-ASIB and SS-FXS). Similarly, average head models were created from the IBIS MRIs based on risk group (IBIS-ASIB and IBIS-FXS). We constructed these four templates using the iterative procedures described in previous publications by Richards and colleagues [53,54,56]. The templates were created separately for the whole head and the extracted brain. The first step was to create a preliminary brain or head average. A rigid rotation (FLIRT 6 parameter linear registration and transformation) [69] to the 12-month-old TD template [53] ensured that all images were oriented in the same way prior to averaging. The second step of the iterative procedure consisted of a non-linear registration (ANTS, “Advanced Normalization Tools”) [70,71] to the current reference average, a transformation of each participant MRI into the template space, and then averaging of the transformed MRIs. This average was then used as the reference model in the next iteration. The root mean square difference between successive average reference models was calculated, and the iterative procedure was carried out until leveling of the successive root mean square values was obtained. The final reference model is the template. For more details and figures depicting this process see Sanchez et al. [53,54].

We also created head models based on average MRI templates from the “Neurodevelopmental MRI Database” [58] as comparisons for the other head models. These included templates created from 12-month-old typically developing (TD) infants (12-mo-TD), 12-year-old TD children (12-yr-TD), and 20- to 24-year-old TD adults (Adult-TD). The templates are continuously updated as MRIs are acquired and currently include structural MRIs collected from 169 12-month-olds, 139 12-year-olds, and 632 20- to 24-year-olds.

#### 2.3.3. Electrode Placement

The EGI Hydrocel Geodesic Sensor Net 128-electrode locations were first computed on the average MRI templates from the Neurodevelopmental MRI Database. We followed a common procedure for estimating electrodes for the average template head models [58]. The electrode locations for 12-month-old infants were determined by taking photographs of the EEG net on the participant’s head and matching electrodes associated with fiducial locations with fiducials on the average MRI templates. Remaining electrode locations were estimated based on their connections to the fiducial electrodes on the EGI Hydrocel Geodesic Sensor Net. Electrode positions for the 12-year-old and adult average templates were derived from measurements with the Geodesic Photogrammetry System. This system digitizes photos of the nets and converts these to electrode placements. The electrode placements were co-registered to the MRIs, averaged, and placed on the corresponding average MRI templates.

The electrode placements for participants in the current study were created in the same manner as those for the 12-month average template. Electrode placements for the study-specific templates (SS-ASIB and SS-FXS) came from averaging the placements from the subjects separately for those two groups and fitting the electrodes to the average template. The electrode placements for the IBIS group templates came from the corresponding study-specific templates by fitting the study-specific template electrode placements on the group templates.

#### 2.3.4. Regions of Interest

**Figure 2:**
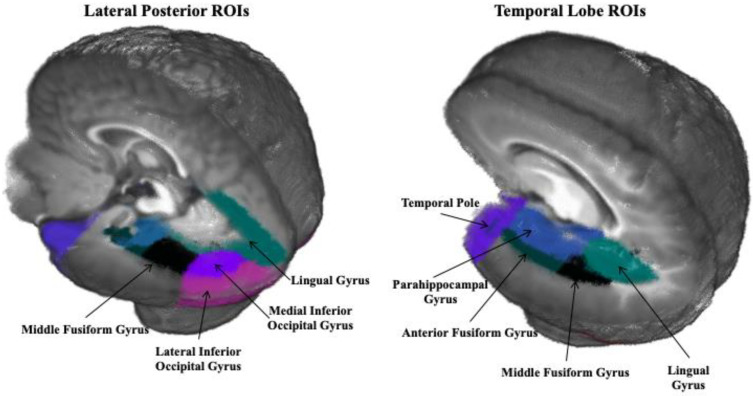
Regions of interest (ROIs) shown on a 6-month-old average age-appropriate template (Guy, Zieber, and Richards, 2016 [13]).

Regions of interest (ROIs) were selected based on those included in recent studies investigating neural sources of the N290 [12,13]. They include the dorsal anterior cingulate gyrus, frontal pole, anterior fusiform gyrus, middle fusiform gyrus, lateral inferior occipital gyrus, medial inferior occipital gyrus, lingual gyrus, middle occipital lobe, middle temporal gyrus, orbital frontal gyrus, parahippocampal gyrus, posterior cingulate gyrus, superior occipital lobe, superior parietal lobe, superior temporal gyrus, superior temporal sulcus, temporal pole, and ventral anterior cingulate. Figure 2 presents several ROIs selected based on proximity to areas identified as sources of the N290, with a few select distant ROIs included as control areas.

### 2.4. Data Analysis

#### 2.4.1. Head Model Comparison

The similarity of the source analysis results from head models created from high-risk infants’ own structural MRIs (Self) were compared against: (a) average head models created from same-group study-specific MRIs (SS-ASIB or SS-FXS); (b) average head models created from same-group IBIS MRIs (IBIS-ASIB or IBIS-FXS); (c) an average head model created from 12-month-old typically developing infants at low risk for ASD (12-mo-TD); (d) an average head model created from 12-year-old children with no known neurodevelopmental disorders (12-yr-TD); and (e) an average head model created from 20–24-year-old adults with no known neurodevelopmental disorders (Adult-TD). Average head models from typically developing groups were acquired from the Neurodevelopmental MRI Database. ANOVAs were calculated to examine differences in the level of activation across ROIs and head models. Pearson correlations were used to examine relatedness in patterns of activation across ROIs and head models. The current density reconstruction (CDR) from the ROIs were examined as dependent variables, and the ROI data from each participant MRI was correlated with the ROI data from the average template head models. Strong positive correlations would indicate a high level of similarity in patterns of activation across head models.

#### 2.4.2. Cortical Source Analysis

Cortical source analysis of the N290 ERP data was completed using the participant’s own MRI head models (when available) and best substitute (when the Self head model was not available). The N290 peak was individually identified for each stimulus type for each participant at electrodes near the parietal–occipital electrodes (PO7: 59, 65, 66; PO8: 84, 90, 91; PO9: 64, 65, 68, 69; PO10: 89, 90, 94, 95), parietal (P7: 51, 58, 59; P8: 91, 96, 97; P9: 57, 58, 63, 64; P10: 95, 96, 99, 100), and temporal–parietal (TP7: 46, 50, 51; TP8: 97, 101, 102; TP9: 50, 56, 57; TP10: 100, 101, 107) electrodes.

**Figure 3:**
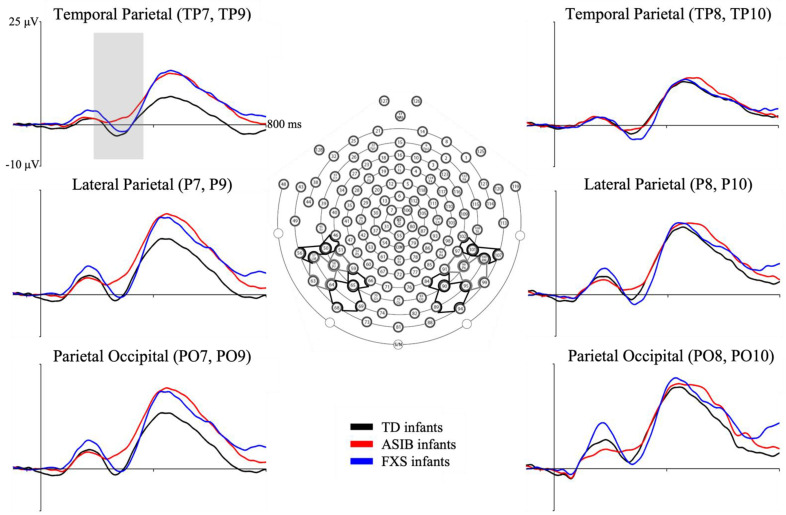
Grand average N290 responses by group at left and right parietal–occipital (PO; left: PO7 and PO9, right: PO8 and PO10), lateral–parietal (P; left: P7 and P9, right: P8 and P10), and temporal–parietal (TP; left: TP7 and TP9, right: TP8 and TP10) electrode clusters. Inferior posterior electrode clusters of interest are presented on the diagram of the electrode layout for the Electrical Geodesics, Inc. (EGI) Hydrocel Geodesic Sensor Net. This figure was first published in Guy, Richards, Tonnsen, and Roberts (2018) [4]. Note. TD = typical development, ASIB = infant sibling of child with autism, FXS = fragile X syndrome.

These electrode clusters are illustrated in Figure 3. A custom MATLAB script was used to identify the point of greatest amplitude for the N290 for each stimulus condition see [72]. The program was constrained to examine peak amplitude between the latency of the P1 peak and 400 ms. Each peak was inspected after detection and misidentified peaks were manually adjusted. If an accurate peak could not be detected by the program or the reviewer, the activity at 290 ms after stimulus onset was examined. Peak amplitude N290 was calculated as the change in amplitude from the peak of the P1 to the peak of the N290. The Fieldtrip toolbox within MATLAB was utilized to estimate cortical sources with current density reconstruction (CDR) [73] using eLORETA [74] as the constraint for the CDR. The CDR was calculated during the 20 ms surrounding the individually identified N290 peaks. Results of the CDR provide information about the current amplitude within each voxel of the realistic head model within the ERP time window of interest. The CDR was summed over each voxel of the ROI and divided by the total volume of the ROI to result in an average current per millimeter value for each ROI.

## 3. Results

### 3.1. N290 ERP Component

**Figure 4:**
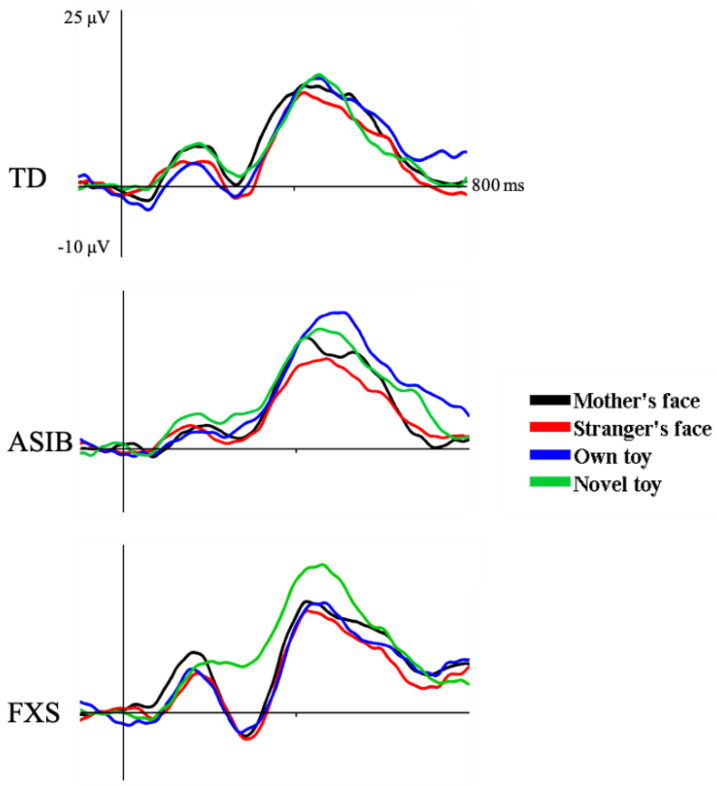
The N290 in response to the mother’s face, a stranger’s face, infant’s own toy, and novel toys separately for each participant group.

The N290 was observed as a negative-going peak in the ERP, occurring approximately 300 ms after stimulus onset. Figure 3 depicts N290 responses at the electrode clusters for each participant group. Figure 4 presents N290 responses separately for each stimulus type averaged across all electrodes analyzed for each participant group. There was a significant main effect of stimulus type. The N290 responses to faces were greater than responses to toys. Although the difference in responses to faces and toys was greatest in infants with FXS, the interaction between participant group and stimulus type was not significant. There was a marginally significant interaction between participant group and stimulus familiarity. This was due to significantly greater N290 amplitude in response to familiar stimuli in infants with FXS compared with participants from the ASIB and LRC groups and compared with all groups’ responses to novel stimuli. Only infants with FXS differentiated stimuli based upon stimulus familiarity (see 4 for additional discussion of these results).

### 3.2. Head Model Comparison

We examined relations in CDR activation between head models created from participants’ own MRIs, IBIS group-specific MRIs, study- and group-specific (i.e., from all ASIBs or infants with FXS that contributed MRIs to the current study), 12-month-old TD infants, 12-year-old TD children, and 20–24 TD adults. A group (2: ASIB, FXS) by head model (6: Self, IBIS, study-specific, 12-Month TD, 12-Year TD, Adult TD) by ROI (18: dorsal anterior cingulate gyrus, frontal pole, anterior fusiform gyrus, middle fusiform gyrus, lateral inferior occipital gyrus, medial inferior occipital gyrus, lingual gyrus, middle occipital lobe, middle temporal gyrus, orbital frontal gyrus, parahippocampal gyrus, posterior cingulate gyrus, superior occipital lobe, superior parietal lobe, superior temporal gyrus, superior temporal sulcus, temporal pole, and ventral anterior cingulate) ANOVA was carried out.

Table 2 presents CDR activation to faces and toys as a function of head model. The CDR activation was most similar between the Self and IBIS head models. There was a main effect of head model, *F* (5, 85) = 15.95, *p* < 0.001, such that CDR activation was most similar across the Self and IBIS head models. Additionally, there was a significant head model by stimulus type interaction, *F* (5, 85) = 5.05, *p* < 0.001. Activation was always greater in response to faces than toys, but the degree of differentiation varied. There was a similar amount of activation to faces and toys across the Self and IBIS head models, but not the other head models.

**Figure 5:**
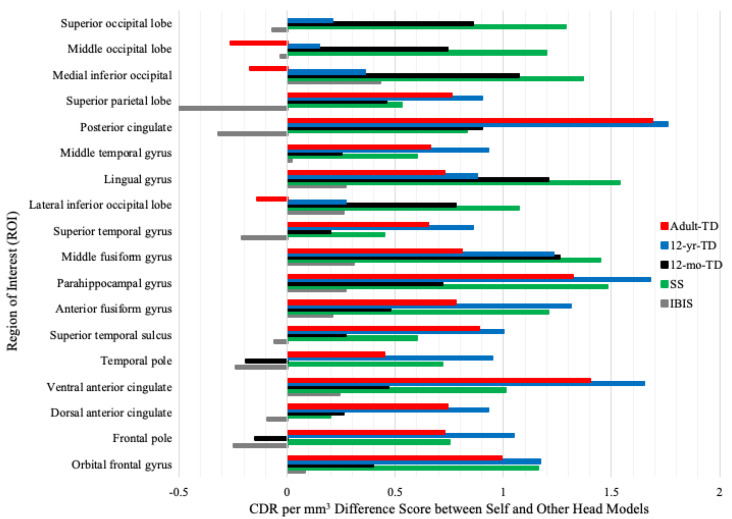
Difference scores calculated by subtracting the CDR for the IBIS, study-specific (SS), 12-mo-TD, 12-yr-TD, and Adult-TD head models from the CDR for the Self model at each ROI.

Figure 5 shows difference scores in CDR activation for the Self head model and other head models across all ROIs examined. Difference scores were lower for the IBIS head models than all other head models. When looking at the difference scores for ROIs related to face processing (i.e., middle and anterior fusiform gyri and parahippocampal gyrus), only the IBIS head models showed a difference score within 0.5 CDR per mm^3^ of the Self head model. There was a head model by ROI interaction, *F* (85, 1445) = 12.41, *p* < 0.001. The Self MRI head model was similar to the IBIS head model in most ROIs (Figure 5). There was also a group by head model by ROI interaction, *F* (85, 1445) = 1.48, *p* = 0.004. ASIBs and infants with FXS had the best fit between the Self and IBIS head models. However, the fit between other head models varied based on etiological group. Table 3 presents the CDR data for the Self, study-specific, and IBIS head models at each ROI sorted by level of activation separately for ASIBs and infants with FXS. The ROIs most closely associated with specialized face processing (i.e., middle and anterior fusiform gyri and parahippocampal gyrus) are highlighted in yellow. These areas are shown to be highly active during the time window of the N290. Highlighted in green are the study-specific or IBIS CDRs most similar to the Self head model. The IBIS head models more closely reflected the Self head model for both ASIBs and infants with FXS, although there was a little variability based on ROI for ASIBs.

We calculated Pearson correlations to examine similarities in activation between the Self MRI head model and all other head models (5: IBIS-ASIB/IBIS-FXS, SS-ASIB/SS-FXS, 12-mo-TD, 12-Yr-TD, and Adult-TD) in response to faces and toys across all ROIs of interest. Table 4 presents correlations between the Self and other head models across and between participant groups. The IBIS head models best correlated with the Self MRI head model. By group, ASIBs showed a very high correlation between the Self head models and the IBIS head model, but infants with FXS showed less strong and consistent correlations.

**Figure 6:**
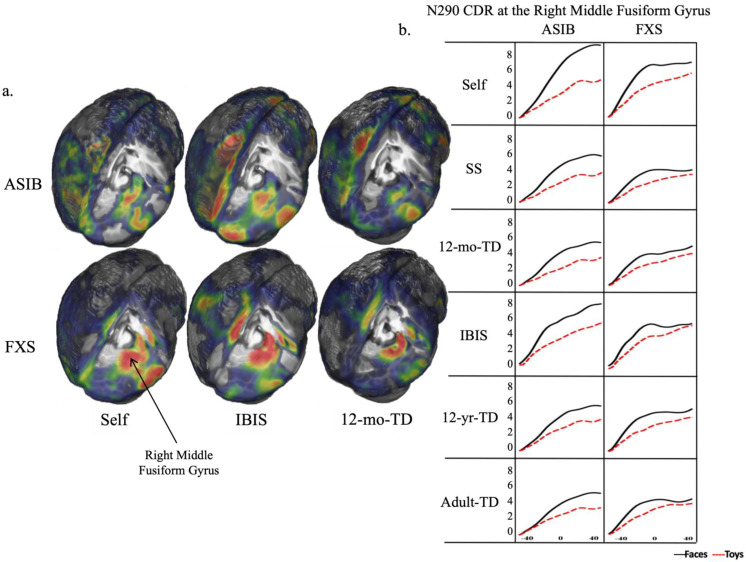
(**a**). Areas and degree of CDR activation are presented on Self, IBIS, and 12-mo-TD whole-head models separately for ASIBs and infants with FXS; warmer colors indicate greater activation; (**b**). Patterns of CDR activation are depicted at the right middle fusiform gyrus during the time window of the N290 separately for ASIBs and infants with FXS for each head model examined.

The results of the analyses comparing head model fit indicate that the IBIS group-specific head models were the best substitution for a head model based on the participant’s own MRI. This was further supported by visual inspection of the data. Figure 6 presents patterns of CDR activation on the whole head at the peak of the N290 and in the right middle fusiform gyrus during the time window of the N290 for each head model examined. Although ROIs were statistically examined bilaterally, Figure 6 provides a detailed examination of activation at the right middle fusiform gyrus. Use of the IBIS head models resulted in patterns of CDR activation most similar in amplitude and peakedness to that of the Self head model. For the ASIBs, increasing activation at the right middle fusiform gyrus is observed across this time window. This is observed using other head models as well. However, only with the IBIS head model do we see a level of activation nearing what was observed with the Self head model. For infants with FXS, there was an increase in activation that resulted in a small peak observed in synchrony with the peak of the N290 ERP response, after which activation appears to stabilize. This pattern of activation, and the level of activation observed, is most closely matched when the IBIS head model is used. This can be seen in the whole-head model, as well as the plots for the middle fusiform gyrus.

### 3.3. Cortical Source Analysis

The cortical source analysis of the N290 was conducted for all participants that contributed EEG data. The individual participant head model was used for infants who had a structural MRI and associated head model. The head model comparison suggests that the IBIS head model was most similar to the participant’s head model for the source analysis results. We completed the cortical source analysis of the N290 using the IBIS head models when the individual MRI head model was not available.

**Figure 7:**
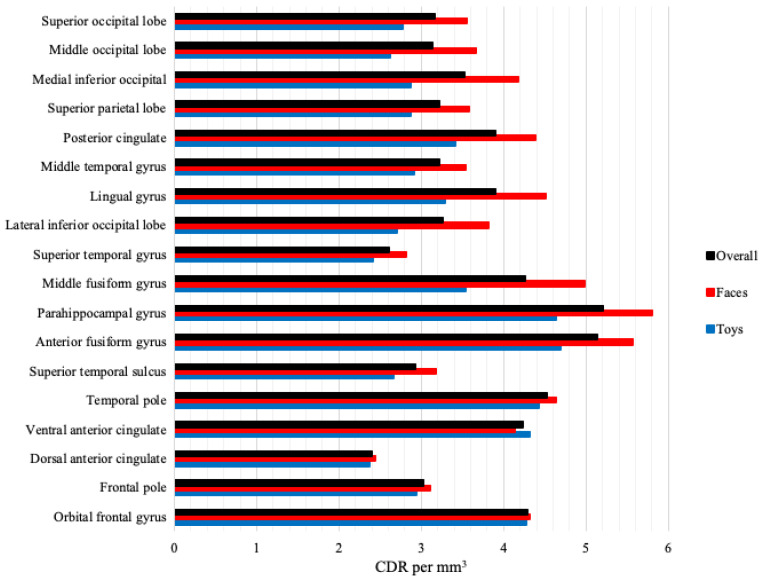
Level of CDR activation is examined across all ROIs as calculated for cortical source analysis using t.

**Figure 8:**
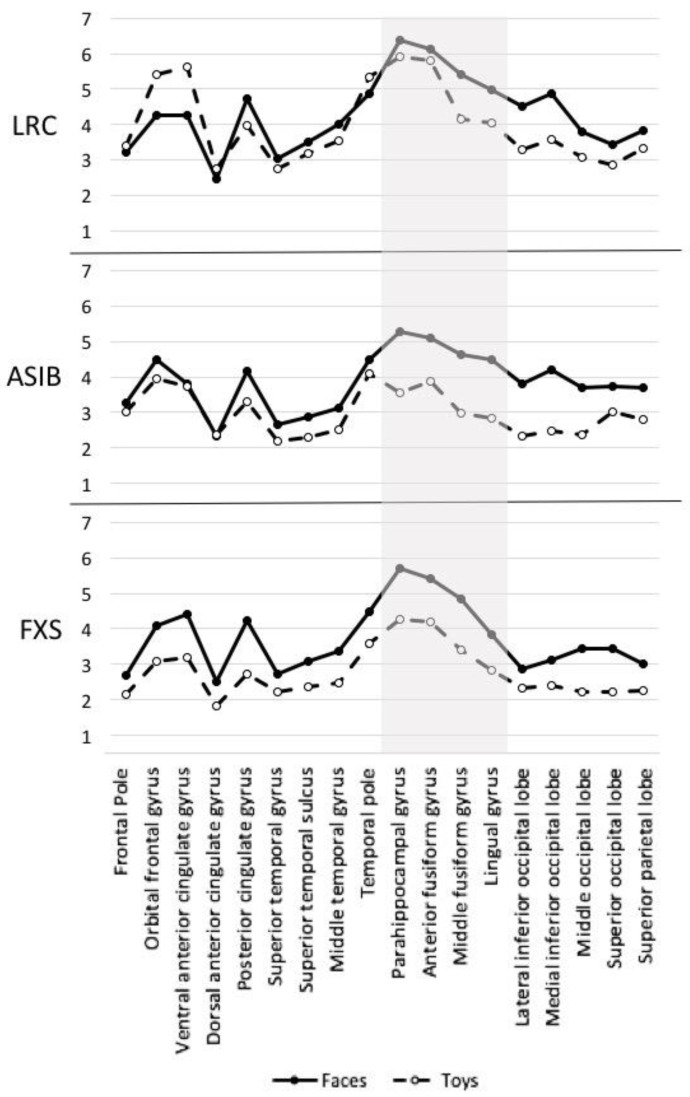
CDR activation to faces and toys is presented for each group and ROI; ROIs associated with the source of the N290 are highlighted in gray.

The source analysis CDR values were analyzed as a function of the stimulus type, group, and ROIs. Figure 7 presents overall CDR activation, as well as CDR to faces and toys, at each ROI examined across participant group. It is apparent that activation was the greatest in the anterior fusiform gyrus and parahippocampal gyrus, but that the greatest differentiation between CDR to faces and toys was observed at the middle fusiform gyrus. These effects were significant. A group (3: ASIB, FXS, LRC) by stimulus type (2: faces, toys) by ROI (18: see above) ANOVA was completed. There was a significant main effect of stimulus type, *F* (1, 55) = 21.47, *p* < 0.001. ROI activation was higher in response to faces (*M* = 4.08) than toys (*M* = 3.31). There was also a main effect of ROI, *F* (17, 935) = 27.67, *p* < 0.001. CDR activation was greatest across groups and stimulus types in the anterior fusiform gyrus (*M* = 5.13) and parahippocampal gyrus (*M* = 5.21). There was a stimulus type by ROI interaction, *F* (17, 935) = 4.91, *p* < 0.001. Activation was greater to faces than toys at all ROIs except for the ventral anterior cingulate. Activation to faces (*M* = 4.98) and toys (*M* = 3.53) was most differentiated at the middle fusiform form gyrus. Finally, there was a significant group by stimulus type by ROI interaction, *F* (34, 935) = 1.45, *p* = 0.047. Figure 8 presents CDR activation to faces and toys separately for each participant group across all ROIs examined. Infants with FXS demonstrated greater activation to faces than toys across all ROIs, and ASIBs demonstrated greater activation to faces at all ROIs except for the dorsal anterior cingulate. LRC infants showed evidence of greater specialization of specific areas for face processing. This was shown in greater activation to faces than toys in most ROIs, but not the dorsal anterior cingulate, frontal pole, orbital frontal gyrus, temporal pole, and ventral anterior cingulate.

## 4. Discussion

### 4.1. Head Model Comparison

Our investigation was based on the assumption that appropriate substitutes for the participants’ own MRIs will be similar in level and patterns of activation. We sought to determine whether specific head models are necessary for accurate source analysis of each high-risk group. We hypothesized that group-specific realistic head models would be most accurate compared with head models created from high-risk infants’ own MRIs. The head models created from MRIs collected from typically developing 12-year-olds and adults were expected to be poor substitutions for head models created from participants’ own MRIs.

The head model comparison included five head models (created from same-group study-specific MRIs, same-group IBIS MRIs, 12-month-old typically developing infant MRIs, 12-year-old typically-developing child MRIs, and typically-developing adult MRIs). These were examined for fit with head models created from participants’ own MRIs. The results of our analyses consistently identify the group-specific IBIS head models as the best substitution for the head model created from the participant’s own structural MRI. As shown in Table 2 and Figure 5 and Figure 6, the use of the IBIS head models resulted in patterns of activation most similar to the Self head model. As is evident in Figure 6, infants with FXS demonstrated a peak in activation at the right middle fusiform gyrus that occurred in synchrony with the peak of the N290 ERP component. This peak was best captured with the IBIS FXS head model. Similarly, the level of activation observed in the CDR plotted with ASIBs’ Self head model was based matched by the IBIS ASIB head model.

It is somewhat surprising that the IBIS head models provided a consistent and considerably better fit than the group- and study-specific head models. One possible explanation is that the larger collection of MRIs utilized for the IBIS head models captures more of the variability in a dataset that is characterized by a high degree of heterogeneity. Brain structures that demonstrate a similar pattern of development would be most accurately represented and may relate to the individual’s structural MRI to a greater extent. ASIBs, in particular, are a very heterogeneous group. They represent many subgroups of individuals affected by, or not affected by, ASD or other outcomes common to ASIBS. Additionally, research examining brain structure in infants at high risk for ASD has identified atypical patterns of brain development. Evidence of overgrowth of the brain in children later diagnosed with ASD has been reported in the first two years of life [59,60,61,62,63]. Sites of the brain that may be most significantly impacted include the middle occipital gyrus, right cuneus, and right lingual gyrus, with additional expansion in the left inferior temporal gyrus and middle frontal gyrus [63]. These results further highlight the need for group-specific head templates for accurate source analysis in infants at high risk for ASD.

The results show that the IBIS head model was the best substitute for infants with FXS, but the effects were less robust. Infants with FXS showed a similar correlation between the Self head model and IBIS, study-specific, and 12-month-old TD infant head models (presented in Table 3). It is possible that there was less heterogeneity in the structure of ROIs that we examined among infants with FXS. That may be more apparent in additional source analyses with other ERP components. Indeed, structural abnormalities have been observed in infants and young children with FXS in past research, e.g., [75,76,77].

The importance of appropriate and specific neuroanatomical head models has been supported by recent work indicating that large-scale studies of brain structure in ASD may be misleading, and that analysis of individuals should occur in more homogenous subgroups to yield the most accurate results, e.g., [78,79,80]. An example of this is seen in a study by Chen and colleagues [78]. Participants in this study were clustered into subgroups based upon neuroanatomical difference patterns observed in imaging data, which produced more accurate results. Furthermore, the current study did not consider ASD outcomes or other clinical characteristics. We recommend the use of the Self head model, when available. However, this work indicates that group-specific head models created from participants of the same age are likely to be a valid substitute when head models from the participants’ own MRIs are not available.

There are limitations to the conclusions that can be drawn from the current analyses. Our hypothesis that the IBIS head models provided a more accurate fit than the study-specific head models due to differences in contributing MRI scans should be investigated empirically. It would be helpful to investigate the reliability of head models derived from varying numbers of individual scans in participant groups expected to be generally homogenous or more heterogeneous in brain structure.

### 4.2. Cortical Source Analysis

This study was the first to conduct cortical source analysis of ERP activity in infants at an elevated risk for autism, and the results provide evidence for unique patterns of neural activation during face processing across infants at high and low risk for ASD and across etiologically distinct high-risk groups. All infants demonstrated greater activation to faces than toys in brain areas most associated with specialized face processing in infancy, including the middle fusiform gyrus, anterior fusiform gyrus, parahippocampal gyrus, and lingual gyrus. This pattern of responses is similar to results recently reported in a cortical source analysis of the N290 in TD infants [12,13]. More widespread, high activation in response to faces in infants with FXS is likely to contribute to the enhanced N290 ERP amplitude displayed by this group. These results indicate that greater amplitude N290 responses observed in infants with FXS are due to an increased area of activation during face processing, rather than greater activation in specialized face processing areas.

Results from the current study contribute to our understanding of the N290 in infants at high risk for ASD. Previous work has shown that distinct groups of infants at high risk for ASD (i.e., ASIBs and infants with FXS) demonstrate unique patterns of N290 activation [4]. This may reflect heterogeneous pathways to social and communicative impairment observed in ASD. Infants with FXS showed evidence of hyper-reactivity based on enhanced responses at the N290 and other ERP components relative to the other participant groups. However, ASIBs’ responses were more characteristic of hypo-reactivity based on more muted ERP responses compared with infants with FXS and LRC infants. Recent work examining relations between infant ERP responses and ASD outcomes in childhood have supported the potential for the N290 as a marker of ASD risk. Shephard and colleagues [18] investigated relations between infant N290 and developmental outcomes in childhood. They found that ASIBs demonstrating greater stimulus-type discrimination characterized by more negative N290 responses to faces than visual noise were more likely to demonstrate atypical N170 lateralization and more severe social-communication impairment at 7 years of age. Different neural sources may be contributing to the enhanced N290 that is associated with emerging symptoms of ASD across groups. Future research is required to better understand relations between infant neural responses and outcome data. We have followed our participants longitudinally. Four of the ASIBs (19%) and five participants with FXS (33%) have received a diagnosis of ASD. These participant groups are too small to examine statistically, however, we hope to supplement this dataset in the future. To better understand cortical sources in relation to outcome data, new head models should be created with diagnosis in mind (i.e., head models from MRIs of ASIBs that receive an ASD diagnosis and head models from MRIs of ASIBs that do not receive an ASD diagnosis).

The resulting head models may serve as a useful tool for the development of measures aiding in early identification of ASD in infancy. The templates created for the current study are available to clinical and experimental researchers. Average templates that have been made available include the T1W head, T2W head, T1W brain, and T2W brain. Age-specific templates are also available as described by Richards and colleagues [53,54,56,58]. The template volumes are stored in NIFTI format on a secure server at nitrc.org. Instructions for access are available online (http://jerlab.psych.sc.edu/neurodevelopmentalmridatabase, accessed on 12 July 2022).

## 5. Conclusions

Results of the current study support the importance of appropriate head models for accurate cortical source analysis. The comparison of available head models for use for cortical source analysis with infant ASIB and FXS ERP data further indicated that unique participant groups warrant unique head models. Etiologically distinct groups of infants at high risk for ASD differ from one another in brain structure and function, and this is an important consideration when determining appropriate head models. The cortical source analysis of the N290 revealed developing face specialization in all participant groups, regardless of risk. This occurred by greater activation to faces than toys in ROIs most closely associated with face processing, including the middle fusiform gyrus. However, subtle differences in activation were seen across high-risk groups, including increased activation to faces across all ROIs examined in infants with FXS and somewhat muted levels of ROI activation in ASIBs. Continued research in this area is important to promoting increased understanding of cortical development associated with social information processing in neurodevelopmental disorders, including FXS and ASD.

## Figures and Tables

**Table 1:** Conductivity values of head model segments.

Segment	Conductivity (S/m)
Scalp	0.3500
Skull	0.0132
Cerebrospinal fluid	1.7900
White matter	0.2000
Gray matter	0.3300
Dura	0.3300
Muscles	0.3500
Eyes	0.5000
Nasal cavity	0.0048

Note. S/m = Siemens per meter.

**Table 2:** Mean CDR amplitude and CDR amplitude in response to faces and toys measured across all ROIs for the Self head model in relation to the other head models (i.e., IBIS, study-specific (SS), 12-mo-TD, 12-yr-TD, and Adult-TD). Negative difference scores indicate lower mean CDR amplitudes than the Self head model.

		CDR per mm^3^		
Head Model	Description	Mean	Faces	Toys
Self	Created from the participant’s own MRI	3.39	3.97	2.80
IBIS-ASIB/IBIS-FXS	Created from 53 (ASIBs) or 24 (FXS) MRIs collected as part of the IBIS study	−0.05	−0.08	−0.01
SS-ASIB/SS-FXS	Created from 8 (ASIBs) or 11 (FXS) MRIs collected as part of the current study	−1.02	−0.63	−0.81
12-mo-TD	Created from 169 MRIs collected for the Neurodevelopmental MRI Database	−0.59	−0.76	−0.42
12-yr-TD	Created from 139 MRIs collected for the Neurodevelopmental MRI Database	−0.91	−1.12	−0.68
Adult-TD	Created from 632 MRIs collected for the Neurodevelopmental MRI Database	−0.60	−0.77	−0.41

Note. ASIB = infant siblings of children with autism; CDR = current density reconstruction; FXS = fragile X syndrome; IBIS = infant brain imaging study; MRI = magnetic resonance image; SS = study-specific; TD = typically developing.

**Table 3:** Mean CDR amplitude separately for ASIBs and infants with FXS at each ROI as calculated using the Self, study-specific (SS), and IBIS head models. Yellow highlights indicate ROIs identified as the sources of the N290 in research with typically developing infants. Green highlights indicate the head model (i.e., study-specific or IBIS) closest in CDR activation to the Self head model at each ROI.

ASIB				FXS			
	Head Model				Head Model		
ROI	Self	SS	IBIS	ROI	Self	SS	IBIS
Parahippocampal gyrus	4.65	3.53	4.54	Parahippocampal gyrus	5.19	3.45	4.80
Anterior fusiform	4.42	3.77	4.49	Anterior fusiform	4.99	3.37	4.57
Lingual gyrus	4.35	2.56	4.24	Middle fusiform	4.33	2.81	3.74
Middle fusiform	4.08	2.71	4.14	Temporal pole	4.24	3.00	4.46
Posterior cingulate	3.78	2.80	4.13	Ventral anterior cingulate	3.94	2.51	3.45
Medial inferior occipital	3.63	2.11	3.46	Orbital frontal gyrus	3.88	2.40	3.61
Superior occipital lobe	3.53	2.44	3.88	Lingual gyrus	3.63	2.27	3.24
Temporal pole	3.50	3.50	3.78	Posterior cingulate	3.46	2.75	3.76
Orbital frontal gyrus	3.26	2.55	3.44	Medial inferior occipital	3.03	1.77	2.41
Superior parietal lobe	3.20	2.94	3.91	Middle occipital lobe	2.97	1.52	2.69
Lateral inferior occipital	3.05	2.02	3.05	Middle temporal gyrus	2.82	2.08	2.75
Middle occipital lobe	3.05	2.18	3.50	Superior occipital lobe	2.82	1.39	2.70
Ventral anterior cingulate	3.03	2.59	3.13	Lateral inferior occipital	2.80	1.70	2.35
Middle temporal gyrus	2.65	2.25	2.70	Superior temporal sulcus	2.60	1.94	2.68
Superior temporal sulcus	2.53	2.02	2.57	Frontal pole	2.57	1.46	2.61
Superior temporal gyrus	2.53	2.02	2.57	Superior parietal lobe	2.53	1.79	2.89
Frontal pole	3.50	3.50	3.78	Superior temporal gyrus	2.37	1.85	2.61
Dorsal anterior cingulate	3.03	2.59	3.13	Dorsal anterior cingulate	2.08	1.93	2.08

Note. CDR = current density reconstruction; ROI = regions of interest; IBIS = Infant Brain Imaging Study; SS = study-specific.

**Table 4:** Pearson correlations between CDR activation measured using the Self head model and average-template-derived head models.

	Correlation with Self Head Model (SD)		
Head Model	Overall	ASIB	FXS
IBIS-ASIB/IBIS-FXS	.84 (.12)	.88 (.07)	.81 (.14)
SS-ASIB/SS-FXS	.81 (.13)	.81 (.17)	.81 (.10)
12-mo-TD	.81 (.15)	.78 (.18)	.82 (.12)
12-yr-TD	.70 (.12)	.77 (.12)	.66 (.26)
Adult-TD	.74 (.18)	.78 (.12)	.72 (.22)

Note. CDR = current density reconstruction; IBIS = Infant Brain Imaging Study; SS = study-specific; TD = typically developing; SD = standard deviation.

## Data Availability

The templates created for the current study are available to clinical and experimental researchers. Average templates that have been made available include the T1W head, T2W head, T1W brain, and T2W brain. Age-specific templates are also available. The template volumes are stored in NIFTI format on nitrc.org. Instructions for access are available online (http://jerlab.psych.sc.edu/neurodevelopmentalmridatabase, accessed on 12 July 2022) and John E. Richards should be contacted regarding interest (richards-john@sc.edu). The ERP data for the study are available on the National Database for Autism Research (NDAR, https://nda.nih.gov, accessed on 12 July 2022).

## Abbreviations

ASD	autism spectrum disorder
CDR	current density reconstruction
ERP	event-related potential
FEM	finite element model
FXS	fragile X syndrome
IBIS	Infant Brain Imaging Study
ASIB	infant siblings of children with autism
LRC	low-risk control
MNI	Montreal Neurological Institute
Nc	Negative central
ROI	region of interest
SS	study-specific
TD	typically developing
